# Selection and validation of reliable reference genes for quantitative real-time PCR in Barnyard millet (*Echinochloa* spp.) under varied abiotic stress conditions

**DOI:** 10.1038/s41598-023-40526-6

**Published:** 2023-09-20

**Authors:** Vellaichamy Gandhimeyyan Renganathan, Raman Renuka, Chockalingam Vanniarajan, Muthurajan Raveendran, Allimuthu Elangovan

**Affiliations:** 1 Department of Biotechnology, Centre of Excellence for Innovations, Agricultural College & Research Institute, Tamil Nadu Agricultural University, Madurai, India; 2 Anbil Dharmalingam Agricultural College & Research Institute, Tamil Nadu Agricultural University, Tiruchirappalli, India; 3 Department of Plant Biotechnology, Centre for Plant Molecular Biology and Biotechnology, Tamil Nadu Agricultural University, Coimbatore, India

**Keywords:** Biotechnology, Plant sciences

## Abstract

Quantitative real-time polymerase chain reaction (RT-qPCR) using a stable reference gene is widely used for gene expression research. Barnyard millet (*Echinochloa* spp.) is an ancient crop in Asia and Africa that is widely cultivated for food and fodder. It thrives well under drought, salinity, cold, and heat environmental conditions, besides adapting to any soil type. To date, there are no gene expression studies performed to identify the potential candidate gene responsible for stress response in barnyard millet, due to lack of reference gene. Here, 10 candidate reference genes, *Actin* (*ACT*)*, α-tubulin* (*α-TUB*)*, β-tubulin* (*β-TUB*)*, RNA pol* II (*RP* II)*, elongation factor-*1 *alpha* (*EF-1α*)*, adenine phosphoribosyltransferase* (*APRT*)*,* TATA*-binding protein-like factor* (*TLF*)*, ubiquitin-conjugating enzyme* 2 (*UBC2*)*, ubiquitin-conjugating enzyme* E2L5 (*UBC5*) and *glyceraldehyde-*3*-phosphate dehydrogenase* (*GAPDH*), were selected from mRNA sequences of *E*. *crus-galli* and *E. colona* var *frumentacea*. Five statistical algorithms (geNorm, NormFinder, BestKeeper, ΔCt, and RefFinder) were applied to determine the expression stabilities of these genes in barnyard millet grown under four different abiotic stress (drought, salinity, cold and heat) exposed at different time points. The *UBC5* and ɑ-TUB in drought, *GAPDH* in salinity, *GAPDH* and *APRT* in cold, and *EF-1α* and *RP* II in heat were the most stable reference genes, whereas *ß-TUB* was the least stable irrespective of stress conditions applied. Further Vn/Vn + 1 analysis revealed two reference genes were sufficient to normalize gene expression across all sample sets. The suitability of identified reference genes was validated with *Cu-ZnSOD* (*SOD1*) in the plants exposed to different abiotic stress conditions. The results revealed that the relative quantification of the *SOD1* gene varied according to reference genes and the number of reference genes used, thus highlighting the importance of the choice of a reference gene in such experiments. This study provides a foundational framework for standardizing RT-qPCR analyses, enabling accurate gene expression profiling in barnyard millet.

## Introduction

Barnyard millet [*Echinochloa frumentacea* (Roxb.) Link; syn. *E. colona* var. *frumentacea*] is a small-seeded, grassy, annual, hexaploid (2n = 6x = 54) minor millet species that survive well even in edaphic environments of temperate and semi-arid tropics of the world. The cultivated *Echinochloa* species of Indian and Japanese barnyard millet are distributed from cold and mountainous areas of Japan to warmer and semi-arid zones (Deccan plateau) of India^1^. In some countries viz., United States and Australia, barnyard millet is grown as a multi-purpose crop to meet the feed and fodder demands of animals due to its high fiber and nitrogen content and good palatable characteristics^2^. Barnyard millet species is a short-duration crop that can grow with almost no input and can withstand various biotic and abiotic stresses^3^. Additionally, this species is well known for phytoextraction due to its ability to hyper-accumulate heavy metals and therefore it has been recommended for soil health restoration programs to reclaim heavy metal-polluted soils^4^. *Echinochloa* species exhibit a high degree of tolerance to both flooding and drought stress due to some specialized rhizosphere structure in maintaining the oxygen level in the plant^1,5,6^.

Besides agronomic advantages, several health benefits of barnyard millet to humankind are reported on consumption, including maintaining blood glucose levels, serum cholesterol, antioxidant activity, anti-inflammatory and in alleviating anemic and constipation-associated diseases^1^. However, the crop is still categorized under the status of neglected and underutilized crop due to a lack of awareness among farmers and the scientific community. The comprehensive research on genetics and genomics is in a preliminary state and is however required for effective utilization of germplasm resources and their subsequent discovery of QTL/gene(s) for important traits. Very recently, the genome of cultivated barnyard millet (*E. frumentacea*) and wild (*Echinochloa* spp.) plants has been sequenced using the diploid-assisted scaffolding method DipHic to better understand the evolution and adaptation of *Echinochloa* species as a weed and as a cultivated crop^7^. However, identification and characterization of genes and pathways associated with resistance to drought, salinity, cold, and flooding stress in *Echinochloa* would be useful to develop superior cultivars and also assist in improving the tolerance in major cereal crops.

Understanding the complex regulatory mechanism involved in the expression of genes requires highly sensitive and accurate instrumentation that quantifies the variable transcriptomes or differentially expressed gene(s) under varying experimental conditions. Quantitative Real-time PCR (RT-qPCR) is an effective tool used to analyze the expression of target genes in different tissues, organs, and environmental conditions^8–10^. It has many advantages over conventional techniques like microarray and Northern blot in terms of rapidity, sensitivity, and reproducibility in synchronized quantification of transcriptional abundance in different samples for a number of genes at a time^8,9,11,12^. Despite the fact that the RT-qPCR offers precise quantification of the gene expression changes, it is easily affected by certain factors, such as quality (RNA integrity) and quantity (concentration) of RNA samples, efficiency of enzymes (DNA polymerase/reverse transcriptase), the specificity and efficiency of primers and the overall transcriptional activity of the cells or tissues analyzed^8,13^. Although numerous normalization approaches are available to overcome these pitfalls in RT-qPCR analysis, the use of internal control or reference gene is the common strategy that is being widely applied across biological systems^14–16^.

Ideal reference genes are usually housekeeping genes that are known to have stable expression across developmental stages, biological conditions, tissues and experimental conditions^14,17^. Therefore, normalization using stable reference genes in transcriptome quantitation is the critical step in RT-qPCR experiments to obtain accurate expression data of the target gene^9,15,18^. Numerous house-keeping genes, such as *actin*, *tubulin*, *18S ribosom*al RNA, *cyclophilin*, *elongation factor-1α*, *glyceraldehyde*-3-*phosphate dehydrogenase*, *ubiquitin*, and *ribosomal protein* L are the most reported reference genes for many plants species^8,19,20^. Nevertheless, several studies have proved that the expression profiles of these traditional reference genes vary under different experimental conditions^21–25^. Moreover, there is no universal reference gene available for all experimental subjects. Therefore, selecting an appropriate ideal reference gene for an appropriate experiment before performing RT-qPCR for any target gene is essential. Numerous platforms which run on various algorithms are available to analyse the stability value of reference genes (ideal gene), including geNorm^26^, NormFinder^27^, BestKeeper^28^, ΔCt^29^ and RefFinder^30^. However, the integrated application of all these software tools increases the accuracy of prediction^31,32^. To date, no such studies or even reference genes are reported for transcript normalization in barnyard millet. Therefore, the present study was undertaken with the objective of identification of stable reference genes in barnyard millet subjected to abiotic stress treatments such as 20% Polyethylene glycol (PEG 6000) for drought, 250 mM sodium chloride (NaCl)) for salinity, 4 °C for cold and 41 °C for heat stress. For this analysis, 10 reference genes were selected from the transcriptome data of wild (*E. crus-galli*) and cultivated (*E. colona* var *frumentacea*) genomes of barnyard millet to use these genes in molecular biological studies of abiotic stress-treated popular variety MDU 1. The results of this study are the first to the best of our knowledge and will provide ideal reference genes in relation to the plant’s response to abiotic stress.

## Results

### Selection and expression profiling of reference genes for RT-qPCR experiments

**Figure 1:**
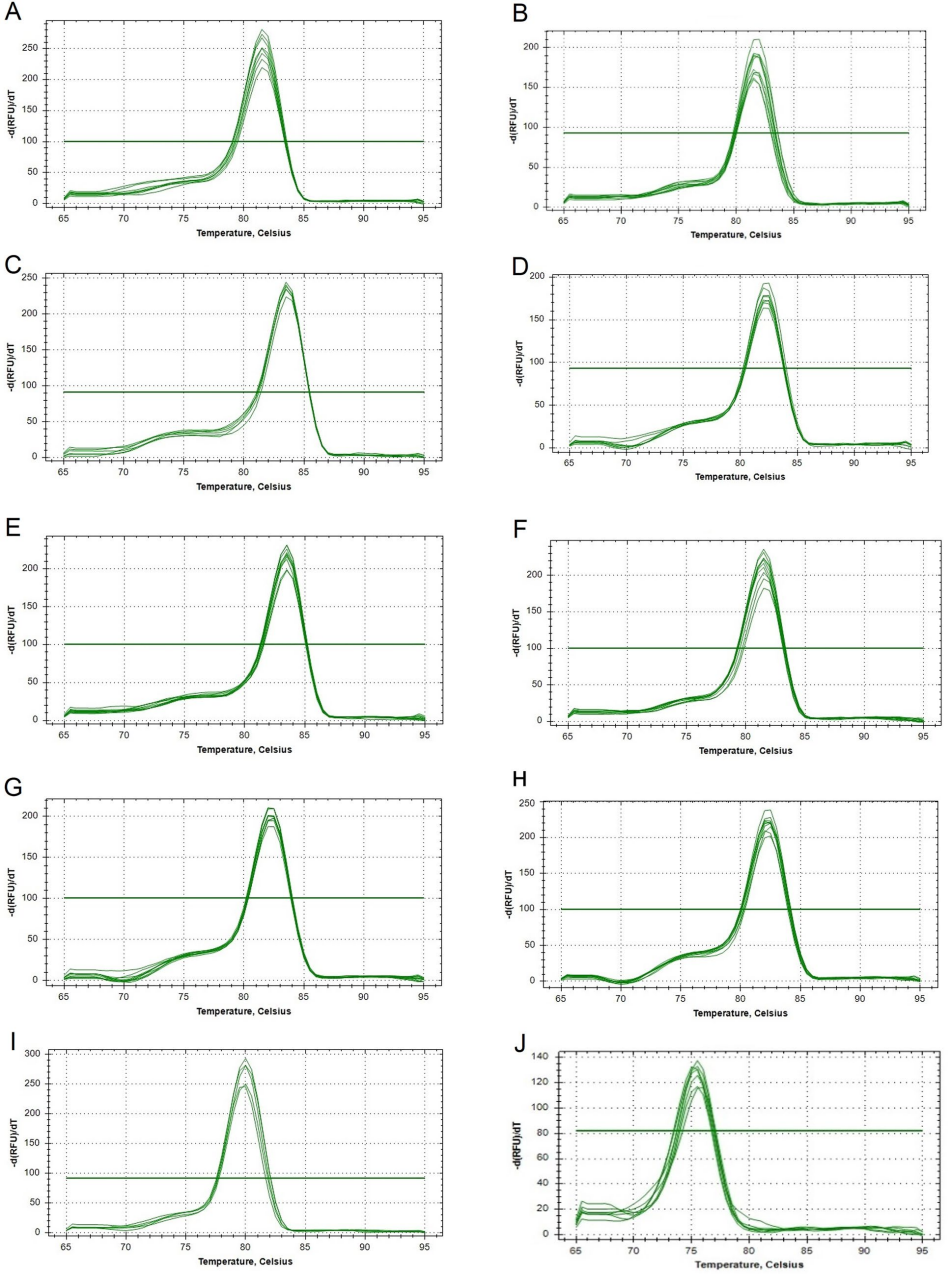
Melt curve analysis of RT-qPCR amplification of ten candidate genes in barnyard millet, (**A**), *Actin*; *ACT* (**B**), *RNA pol* II; *RP* II (**C**), *α-tubulin*; *α-TUB* (**D**), *β-tubulin*; *β-TUB* (**E**), *elongation factor-*1 *alpha*; *EF-1α* (**F**), *adenine phosphoribosyltransferase*; *APRT* (**G**), TATA*-binding protein-like factor*; *TLF* (**H**), *ubiquitin-conjugating enzyme* 2; *UBC2* (**I**), *ubiquitin-conjugating enzyme* E2L5; *UBC5* and (**J**) *glyceraldehyde-*3*-phosphate dehydrogenase*; *GAPDH*.

To identify the stable reference genes, CDS sequences of *E. crus-galli* and *E. colona* var *frumentacea* were used (Supplementary Table S1). The target gene was selected from the CDS of *E. frumentacea* for RT-qPCR validation (Supplementary Table S1). The details of the genomic location of gene sequences, primer sequences, amplicon size, melting temperature (Tm), amplification efficiencies and linear regression (R^2^) values are presented in Supplementary Table S2. Agarose gel electrophoresis (2.0%) and melting curve analysis (RT-qPCR) of each gene depicted the expected amplicon length and single amplification peak, which confirmed the specificity of the primers (Fig. 1). Moreover, no signal was detected in the NTC (no template control) samples, ensuring the absence of contamination of reagents. The efficiencies of respective primers were substantiated with linear regression (R^2^), and amplification efficiency calculation (E values, %), where the R^2^ values were more than 0.97 and E values ranged between 91% (*GAPDH*) and 110% (*ɑ-TUB*) (Supplementary Table S2).

**Figure 2:**
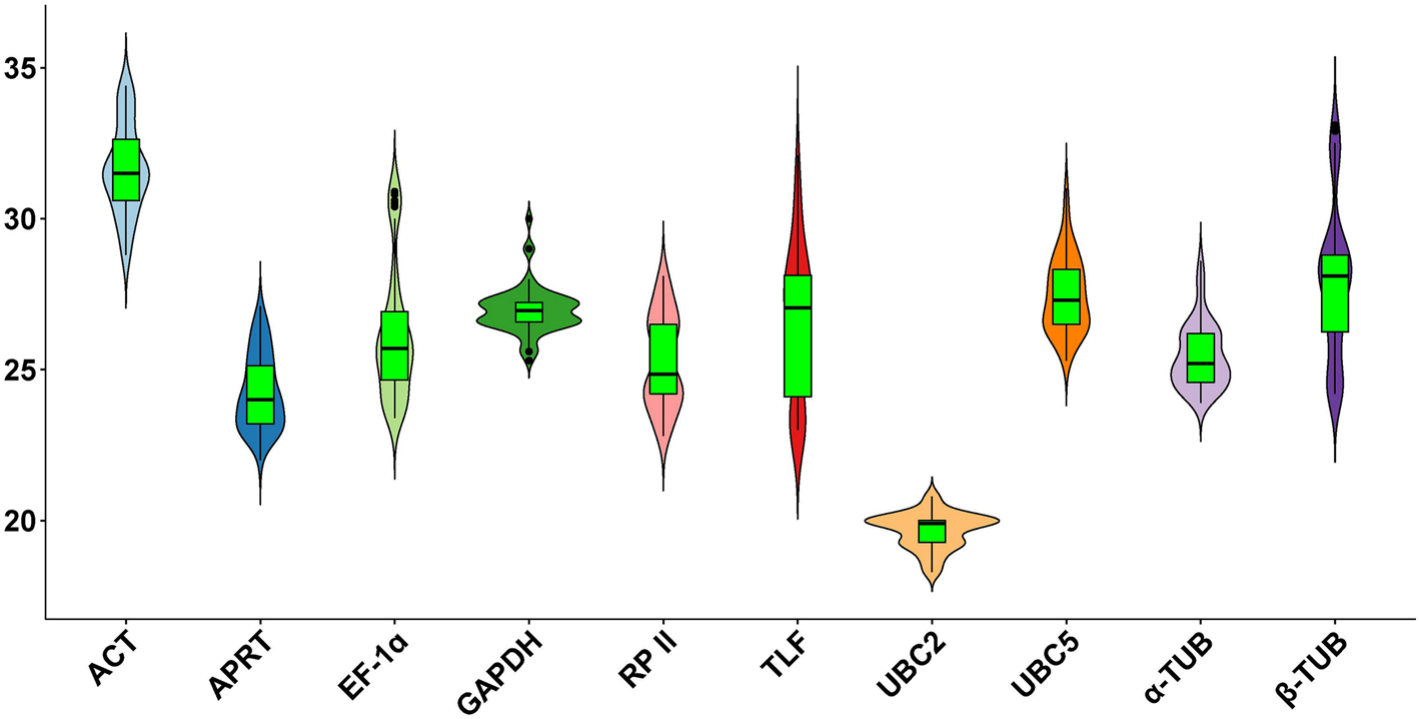
RNA expression profiles of candidate reference genes (*Actin* (*ACT*)*, α-tubulin* (*α-TUB*)*, β-tubulin* (*β-TUB*)*, RNA pol* II (*RP* II)*, elongation factor-1 alpha* (*EF-1α*)*, adenine phosphoribosyltransferase* (*APRT*)*,* TATA*-binding protein-like factor* (*TLF*)*, ubiquitin-conjugating enzyme* 2 (*UBC2*)*, ubiquitin-conjugating enzyme* E2L5 (*UBC5*) and *glyceraldehyde-*3*-phosphate dehydrogenase* (*GAPDH*) analysed; represented as cycle threshold value (Cq) in the different treatment samples collected 0, 12, 24, 36 and 48 h duration of post stress. The solid line within each box represents the median Cq values, and the Cq values are the mean of three replicates. Black dots represent the potential outliers.

The expression levels of these reference genes were analyzed using Cq (quantification cycle) values of respective cDNA samples of drought (PEG), salinity (NaCl), cold (4 °C), and heat (41 °C) at various time points (Supplementary Fig. S1). The Cq values of the reference gene showed different levels of expression profile under all experimental conditions (Supplementary Table S3, Fig. 2). The mean Cq values varied from 19.3 (*UBC2*) to 34.2 (*ACT*) for drought, 18.6 (*UBC2*) to 32.7 (*ACT*) for salinity, 19.8 (*UBC2*) to 31.5 (*ACT*) for cold and 18.9 (*UBC2*) to 31.9 (*ACT*) for heat-treated samples. As inferred from the values of all the treated samples, *UBC2* had the lowest Cq value indicating the highest level of expression, *ACT* had the highest Cq value indicating the lowest level of expression irrespective of abiotic stresses under study (Fig. 2).

### Expression stability of the reference genes

After a simple comparison of the Cq values, the expression stability of respective genes was further analyzed using independent algorithms viz., GeNorm, NormFinder, Bestkeeper, Delta Ct (ΔCt), and RefFinder methods across all the experimental samples.

### GeNorm analysis

The expression stability of 10 candidate reference genes was carried out using the geNorm platform. The geNorm software analyses datasets and estimates stability based on the M value (stability value). The genes with lower M values were considered to have the most stable expression, whereas those with higher M values were ranked as the least stable. The geNorm suggests M = 0.5 as a cutoff value, where the genes with M values higher than 0.5 should not be used as reference genes^26^. As shown in Table 1, the M value varied from 0.396, 0.375, 0.469, and 0.381 to 2.043, 1.13, 1.27, and 1.10 of respective lowest and highest values of drought, salinity, cold and heat treatments. Based on geNorm software results, the following genes were assessed as most stable candidate references viz., *RP* II and *ɑ-TUB* (drought), *ɑ-TUB* and *GAPDH* (salinity), *APRT* and *UBC2* (cold) and *RP* II and *UBC2* (heat). The rest of the genes showed a low level of expression stability. *TLF*, *ß-TUB*, and *ACT* were the least stable genes in all the environmental conditions.

**Table 1:** Expression stability values (SV) and the rankings calculated using geNorm, NormFinder, BestKeeper, ΔCt and RefFinder.

Experimental conditions	Rank	GeNorm		NormFinder		BestKeeper		ΔCt		RefFinder	
		Gene	SV	Gene	SV	Gene	SV	Gene	SV	Gene	SV
PEG	1	*RP* II	0.396	*APRT*	0.479	*UBC2*	0.284	*UBC5*	1.54	*UBC5*	2.06
	2	*ɑ-TUB*	0.396	*UBC5*	0.660	*GAPDH*	0.397	*APRT*	1.59	*UBC2*	2.78
	3	*UBC5*	0.607	*ACT*	1.005	*UBC5*	0.649	*UBC2*	1.77	*ɑ-TUB*	2.83
	4	*UBC2*	0.705	*ɑ-TUB*	1.335	*ɑ-TUB*	0.662	*ɑ-TUB*	1.79	*APRT*	2.91
	5	*GAPDH*	0.831	*UBC2*	1.340	*RP* II	1.003	*ACT*	1.80	*RP* II	3.96
	6	*APRT*	1.047	*GAPDH*	1.378	*APRT*	1.050	*GAPDH*	1.84	*GAPDH*	4.36
	7	*ACT*	1.148	*RP* II	1.399	*ACT*	1.235	*RP* II	1.85	*ACT*	5.21
	8	*EF-1α*	1.440	*EF-1α*	1.734	*EF-1α*	2.189	*EF-1α*	2.29	*EF-1α*	8.00
	9	*TLF*	1.701	*TLF*	2.094	*TLF*	2.286	*TLF*	2.52	*TLF*	9.00
	10	*ß-TUB*	2.043	*ß-TUB*	3.215	*ß-TUB*	3.467	*ß-TUB*	3.41	*ß-TUB*	10.00
NaCl	1	*ɑ-TUB*	0.375	*ɑ-TUB*	0.146	*GAPDH*	0.323	*ɑ-TUB*	0.86	*ɑ-TUB*	1.41
	2	*GAPDH*	0.375	*EF-1α*	0.364	*UBC5*	0.418	*EF-1α*	0.93	*GAPDH*	1.73
	3	*UBC5*	0.486	*GAPDH*	0.501	*UBC2*	0.475	*GAPDH*	0.94	*EF-1α*	3.16
	4	*RP* II	0.595	*APRT*	0.666	*ɑ-TUB*	0.489	*UBC5*	1.03	*UBC5*	3.31
	5	*EF-1α*	0.674	*UBC5*	0.697	*EF-1α*	0.738	*RP* II	1.07	*RP* II	5.18
	6	*APRT*	0.788	*RP* II	0.720	*RP* II	0.813	*APRT*	1.08	*APRT*	5.63
	7	*UBC2*	0.867	*ACT*	1.099	*APRT*	0.893	*UBC2*	1.29	*UBC2*	5.86
	8	*ACT*	0.942	*UBC2*	1.108	*ACT*	0.922	*ACT*	1.32	*ACT*	7.74
	9	*ß-TUB*	1.047	*ß-TUB*	1.138	*ß-TUB*	1.096	*ß-TUB*	1.35	*ß-TUB*	9.00
	10	*TLF*	1.138	*TLF*	1.353	*TLF*	1.388	*TLF*	1.50	*TLF*	10.00
Cold	1	*APRT*	0.469	*GAPDH*	0.561	*UBC2*	0.585	*GAPDH*	1.07	*GAPDH*	1.73
	2	*UBC2*	0.469	*UBC5*	0.582	*APRT*	0.311	*UBC5*	1.10	*APRT*	2.06
	3	*GAPDH*	0.698	*APRT*	0.608	*GAPDH*	0.717	*APRT*	1.10	*UBC2*	2.21
	4	*UBC5*	0.742	*EF-1α*	0.697	*ACT*	0.721	*UBC2*	1.15	*UBC5*	2.99
	5	*ɑ-TUB*	0.835	*RP* II	0.701	*UBC5*	0.773	*RP* II	1.16	*EF-1α*	5.63
	6	*RP* II	0.927	*UBC2*	0.767	*EF-1α*	0.832	*EF-1α*	1.19	*RP* II	5.89
	7	*EF-1α*	0.981	*ɑ-TUB*	0.785	*ɑ-TUB*	0.972	*ɑ-TUB*	1.20	*ɑ-TUB*	6.44
	8	*ß-TUB*	1.015	*ß-TUB*	0.849	*RP* II	0.974	*ß-TUB*	1.22	*ACT*	7.35
	9	*ACT*	1.125	*ACT*	1.534	*ß-TUB*	1.109	*ACT*	1.67	*ß-TUB*	8.24
	10	*TLF*	1.271	*TLF*	1.741	*TLF*	1.556	*TLF*	1.85	*TLF*	10.00
Heat	1	*RP* II	0.381	*EF-1α*	0.343	*RP* II	0.486	*EF-1α*	0.91	*EF-1α*	2.55
	2	*UBC2*	0.381	*APRT*	0.461	*UBC2*	0.488	*APRT*	0.94	*RP* II	2.65
	3	*ACT*	0.672	*UBC5*	0.511	*GAPDH*	0.501	*UBC5*	0.97	*APRT*	2.83
	4	*APRT*	0.737	*ACT*	0.579	*APRT*	0.576	*ACT*	0.99	*UBC2*	3.36
	5	*UBC5*	0.795	*GAPDH*	0.646	*ACT*	0.583	*GAPDH*	1.03	*ACT*	3.94
	6	*EF-1α*	0.822	*ɑ-TUB*	0.791	*UBC5*	0.623	*ɑ-TUB*	1.13	*UBC5*	4.05
	7	*GAPDH*	0.836	*RP* II	0.953	*EF-1α*	0.797	*RP* II	1.14	*GAPDH*	4.79
	8	*ɑ-TUB*	0.915	*UBC2*	1.041	*ɑ-TUB*	1.149	*UBC2*	1.20	*ɑ-TUB*	6.93
	9	*TLF*	1.013	*TLF*	1.051	*TLF*	1.384	*TLF*	1.25	*TLF*	9.00
	10	*ß-TUB*	1.103	*ß-TUB*	1.350	*ß-TUB*	1.582	*ß-TUB*	1.46	*ß-TUB*	10.00

**Figure 3:**
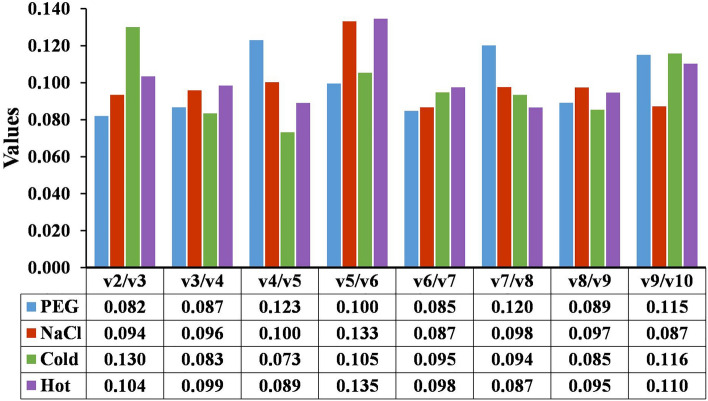
Determination of optimal number of reference genes. Pairwise variation (Vn/n + 1) analysis of 10 candidate reference genes in four different sample sets. The inclusion of an additional reference gene is not required below the cut-off value of 0.15.

To determine the optimal number of reference genes required for accurate normalization, pairwise variations (Vn/Vn + 1) were analyzed based on the normalization factor (NFn and NFn + 1) using geNorm. According to the geNorm algorithm, the cut-off value of pairwise variations (Vn/Vn + 1) of n genes was set at 0.15. It has been suggested that if Vn/Vn + 1 was below 0.15, the additional reference gene (n + 1) is not necessary for reliable normalization. In the results of the four experimental groups analyzed, all experimental sets were below the threshold value of 0.15, indicating that two reference genes are sufficient to normalize gene expression data (Fig. 3).

### NormFinder analysis

NormFinder software calculated the expression stability value based on intra- and inter-group variances of candidate reference genes under study. Similar to geNorm, NormFinder analysis with a higher value indicated lower stability, whereas a lower value indicated greater gene stability^27^. NormFinder analysis showed that *APRT* (0.479) and *UBC5* (0.660) were the two most stable genes under drought stress conditions; *ɑ-TUB* (0.146) and *EF-1ɑ* (0.364) were the two most stable genes under salinity; *GAPDH* (0.561) and *UBC5* (0.582) were the two most stable genes under cold; and *EF-1ɑ* (0.343) and *APRT* (0.461) were the two most stable genes in the heat stress conditions. Although the results of NormFinder and geNorm were slightly different (Table 1), both platforms revealed that the *APRT*, *GAPDH* and *ɑ-TUB* genes were the most stable genes. Notably, *TLF* and *ß-TUB* were the most unstable genes with the highest stability value of 1.05 and 3.02, which is completely consistent with the results determined through geNorm.

### BestKeeper analysis

The expression stability was estimated by BestKeeper software, where the coefficient of determination (r), standard deviation (SD) and coefficient of variation (CV) of each gene with the geometric mean of all genes were considered for final ranking^28^. The most stable reference genes must have the highest r, lowest SD and CV. According to the BestKeeper algorithm (Table 1), the most stable genes are *APRT*, *GAPDH*, *RP* II, and *UBC2* for all experimental conditions. The least stable are *TLF* and *ß-TUB*. These results were consistent from those of NormFinder and geNorm.

### ΔCt analysis method

According to the ΔCt analysis method, the ranking order of candidate reference genes was generated by the SD values (Table 1). The lower the mean SD value, the more stable the expression of the reference gene^29^. ΔCt analysis method (Table 1) shows that *UBC5* (1.54) and *APRT* (1.59) in drought, *ɑ-TUB* (0.86) and *EF-1ɑ* (0.93) in salinity, *GAPDH* (1.07), *UBC5* (1.10) and *APRT* (1.10) in cold and *EF-1ɑ* (0.91) and *APRT* (0.94) were the most stable reference genes under respective stress conditions, while *TLF* (1.25–2.52) and *ß-TUB* (1.22–3.41) showed least stability invariably in all experimental samples. In addition, the most and least stable reference genes identified in different experimental conditions are consistent with the analysis determined using the three other programs geNorm, NormFinder, and BestKeeper with minor changes in the ranking (Table 1).

### RefFinder analysis

**Figure 4:**
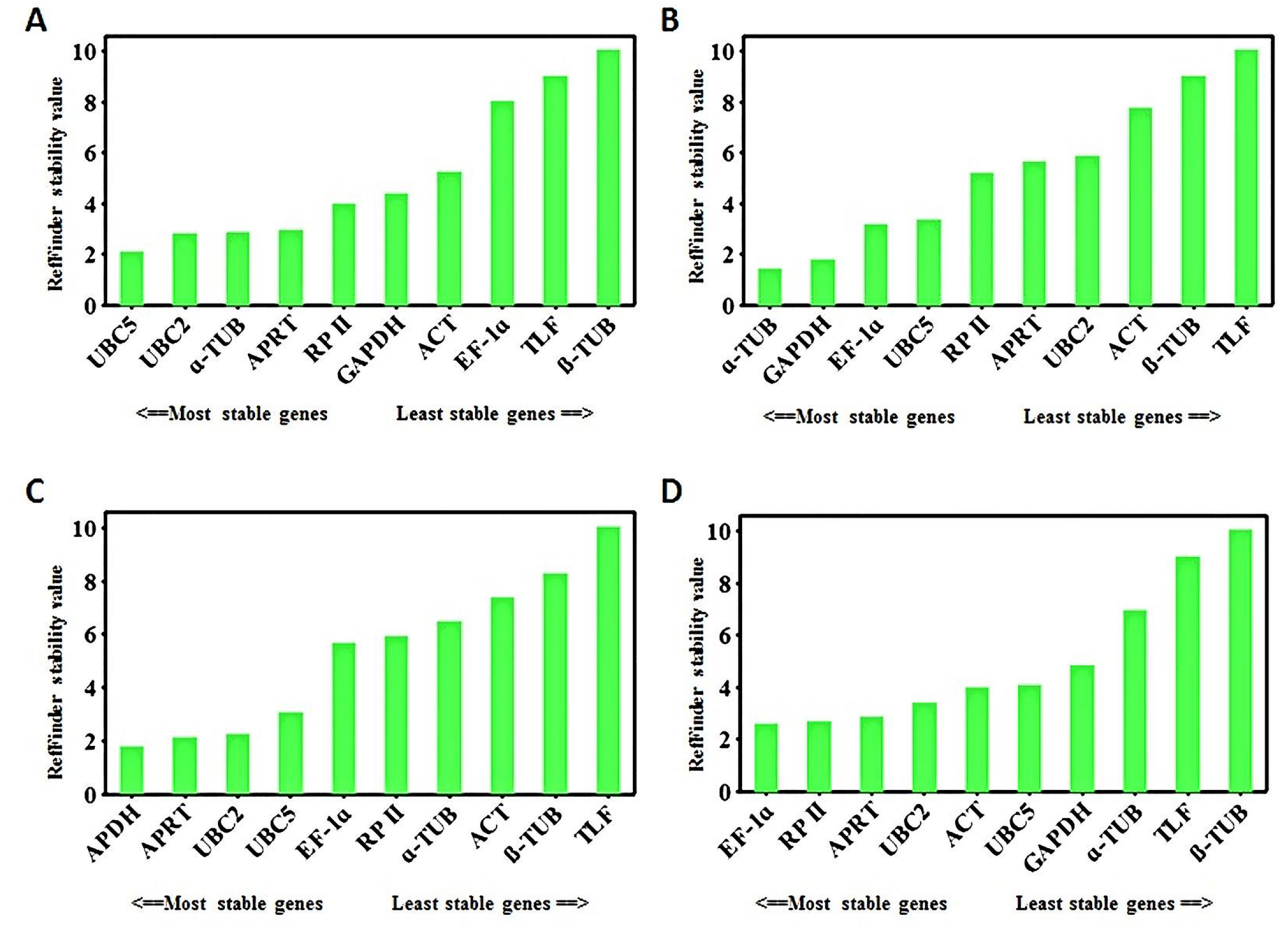
Comprehensive expression stability and ranking of candidate reference genes. *Actin* (*ACT*)*, α-tubulin* (*α-TUB*)*, β-tubulin* (*β-TUB*)*, RNA pol* II (*RP* II)*, elongation factor-*1 *alpha* (*EF-1α*)*, adenine phosphoribosyltransferase* (*APRT*)*,* TATA*-binding protein-like factor* (*TLF*)*, ubiquitin-conjugating enzyme* 2 (*UBC2*)*, ubiquitin-conjugating enzyme* E2L5 (*UBC5*) and *glyceraldehyde-*3*-phosphate dehydrogenase* (*GAPDH*) as calculated by RefFinder in the stress treated samples. (**A**), Drought (**B**), Salinity (**C**) Cold and (**D**) Heat.

The four different algorithms (geNorm, NormFinder, BestKeeper and ΔCt) generated the same or different ranking for the stable reference genes across the experimental conditions. To overall rank the reference genes under different stress conditions, RefFinder was used to comprehensively determine the stability of the candidate reference genes^30^. Based on the rankings from each algorithm, the RefFinder algorithm assigns an appropriate weight to each gene and calculates the geometric mean of their weights for the overall final ranking (Fig. 4). Analysis of all datasets (considering all treatment samples) revealed that the genes *APRT*, *ɑ-TUB*, *RP* II, *UBC2,* and *UBC5* are the most stably expresses genes (top five ranks), with some minor differences in the rankings. Interestingly, in all statistical algorithms, *TLF *and *ß-TUB* were identified to be the least stable genes for abiotic stress conditions.

### Validation of candidate reference genes

To validate the identified stable reference genes under drought, salinity, cold and heat conditions, the expression level of *SOD1* was analyzed for normalization. *SOD1* is a key antioxidant gene that is upregulated in all parts of the cell to protect the plants from harmful reactive oxygen species (ROS) during responses to abiotic stress and thus has a significant impact on growth and development^33,34^,. In the present study, we used the most and least stable reference genes ranked by RefFinder for the normalization of *SOD1* under four different abiotic stress treatments. It includes *UBC5 *and *ɑ-TUB* for drought, *ɑ-TUB* and *GAPDH* for NaCl, *GAPDH* and *APRT* for cold and *EF-1ɑ,* *RP* II and *UBC2* for heat stress, and the least stable reference gene *ß-TUB*.

**Figure 5:**
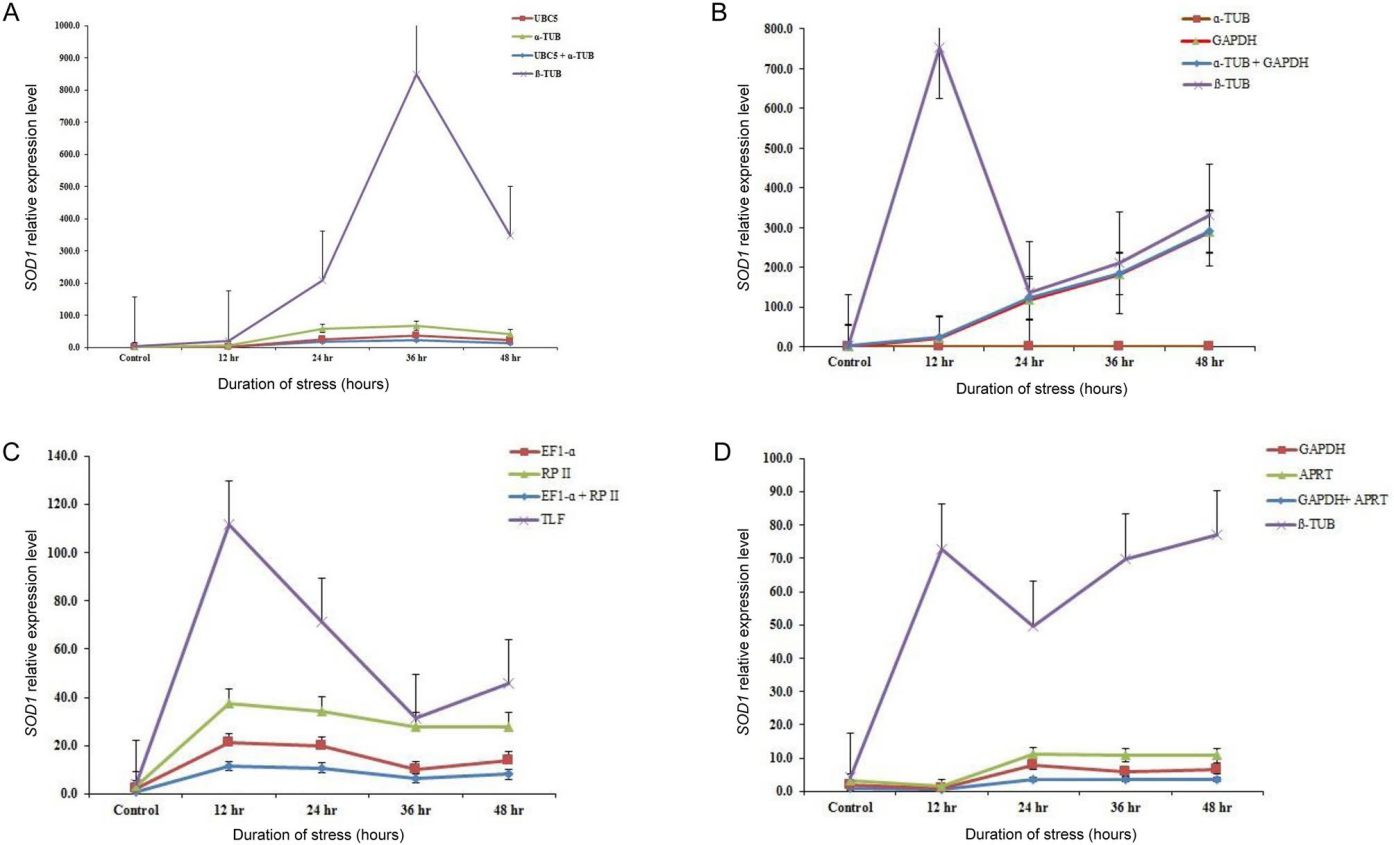
The expression level of the *SOD1* in various abiotic stress sample sets. The respective two top-ranked genes, *ubiquitin-conjugating enzyme* E2L5 (*UBC5*) & *α-tubulin* (*α-TUB*) in drought (**A**), *α-tubulin* (*α-TUB*) & *glyceraldehyde-*3*-phosphate dehydrogenase* (*GAPDH*) in NaCl (**B**), *glyceraldehyde-*3*-phosphate dehydrogenase* (*GAPDH*) & *adenine phosphoribosyltransferase* (*APRT*) in cold (**C**) and *elongation factor-*1 *alpha* (*EF-1α*) & *RNA pol* II (*RP* II) in heat (**D**) were used for normalization as most stable reference genes, *β-tubulin* (*ß-TUB*)/ TATA*-binding protein-like factor* (*TLF*), the last-ranked gene was used as least stable reference gene. Vertical bars indicate the standard deviations (SD).

For PEG treatment, the expression level of *SOD1* showed minor variation when normalized by the most stable two reference genes alone or in combination. However, abnormal expression was documented when normalized by the least (unstable) reference gene *ß-TUB* (Fig. 5A). As shown in Fig. 5A, the normalized expression level of *SOD1* in drought increased gradually at 24 and 36 h and then decreased at 48 h when normalized using the two most stable genes (*UBC5* and *ɑ-TUB*), while the expression level was extremely high at 36 h when *ß-TUB* was used as a reference gene. In response to NaCl stress treatment (Fig. 5B), the expression levels of *SOD1 *was significantly affected when the topmost stable gene *ɑ-TUB* alone was used. However, the expression of *SOD1* was prominently increased, when normalization was done using *ɑ-TUB* along with *GAPDH* (the second most stable gene). Whereas the expression of the least stable reference gene (*ß-TUB*) showed a very high level of expression. For cold stress normalization (Fig. 5C), when the best genes *GAPDH* or *APRT* alone or in combination were used, the expression changes were the same, especially at 12 h the expression decreased and at later hours it increased gradually. When the least stable gene *ß-TUB* was used for normalization, a sustained increase in the expression level of *SOD1* was observed after stress, which exhibited an overestimation of expression when compared with the results of the expression changes of the former two stable genes. In response to heat stress (Fig. 5D), the expression levels of *SOD1* showed a similar trend when normalized using the stable reference gene *EF-1ɑ* alone or in combination with *RP* II. The *SOD1* expression increased continuously after 12 h and then decreased drastically at 36 h and again increased trend of *SOD1* expression was observed at 48 h when the least stable gene (*ß-TUB*) was selected as the reference gene.

In summary, as shown in Fig. 5, stable reference gene alone or when used in combination with the top two stable genes for normalization, the relative expression of *SOD1* displayed a similar trend (gradual increase) in all abiotic stress. However, when the least stable gene *ß-TUB* was used for normalization, the expression patterns were either overestimated or underestimated.

## Discussion

The selection of a suitable reference gene is very important for accurate gene quantification in RT-qPCR studies. The ideal reference gene must have a relatively stable expression in the tested samples irrespective of experimental conditions such as cell types, tissues, developmental stages, growth conditions, etc^35^. However, to date, no such reference genes have been proven to provide stable expression profiles across all investigational conditions throughout the plant and animal kingdom. In recent reports, the most commonly used reference genes including *Actin, tubulin, glyceraldehyde-3-phosphate dehydrogenase,* and *elongation factor 1α,* etc., proved that their transcript levels vary in different plant species under different experimental conditions^36–38^. This might be due to differences in the cell basal metabolism and specific cellular functions and thus have the extensive molecular regulation changes of reference genes of the species under different environmental conditions^14,38–40^. Hence, the reference gene used for one experiment may be unfit for other experimental conditions; even if the same experimental species is used. For example, in Arabidopsis, *Actin* was best stable under both biotic and abiotic stress but least stable under developmental stages, while *UBQ10* appeared to be the most stably expressed gene^39^. The unsatisfactory performance of *Actin* has also been reported under various experimental conditions (developmental stages, tissues, hormones and abiotic stress) in maize^36^ and garlic^37^. Similarly in cotton, under salt stress, the *Actin14, Histone H3 (HIS3)* and *Translation elongation factor 1A-8 (EF1A8) *were most stable in leaves while *a-Tubulin10* and *UBQ7* genes in roots^41^. Therefore, selecting suitable reference genes for normalization studies appropriate to species and experimental conditions is necessary. To date, many literature relating to the reference genes validation under different environmental conditions is being reported in several crop species including Arabidopsis, banana, cucumber, foxtail millet, flax, grape, peanut, potato, safflower, soybean, radish, tomato, tobacco and wheat^24,38,42^. However, to date, no systematic study has been carried out in barnyard millet crops for the validation of reference genes under different experimental conditions. Hence, considering the climate-resilient feature of the crop and stress-specific reference genes for accurate normalization of transcripts in RT-qPCR, the present study was carried out to identify valid reference genes under different abiotic stress conditions.

The study involves 10 candidate reference genes designed from CDS regions of *Echinochloa* species (Supplementary Table S1) that were systematically screened under various abiotic stresses (drought, salinity, cold, and heat) and evaluated for their stability in expression using five different platforms namely, GeNorm^26^, NormFinder^27^, BestKeeper^28^, ΔCt^29^, and RefFinder^30^. Previous to these statistical analyses, all 10 primer pairs were confirmed to fulfill the criteria for E and R^2^ values (Supplementary Table S2) and thus were selected and further utilized in five independent platforms to identify suitable reference genes. The PCR results confirmed that each pair of primers amplified a specific band, and the melt curve analysis also exhibited a single peak. The melt curve analysis of ten candidate genes were depicted in Fig. 1. The E value of the primer was between 91.2 and 110.1% with the R^2^ values between 0.97 and 0.99 (Supplementary Table S2), which indicated their amplification-specificity for further utilization in gene expression analysis.

As shown in Table 1, the 10 reference genes exhibited variable expression stability in response to different stresses. The ranking order of gene stability by five algorithms showed slight differences, which is expected because of their distinct principle for evaluating reference genes^22,24,25,35,43^. However, for all the abiotic stress conditions of this study, geNorm, NormFinder, Best Keeper, and ΔCt consistently ranked the same genes (top-ranked) as the most stable candidate reference genes. Our results were in agreement with the recent reports of Dudziak et al.^24^ and Zhang et al.^38^, who demonstrated that all the used algorithms except Best Keeper produced the same genes (top-ranked) under different abiotic stress conditions in wheat^24^ and *Salix* plants^38^. Interestingly, the ranking order of the most unstable genes identified by the five algorithms was exactly consistent in all sample sets. However, many reports suggested that the integrated analysis using the results of independent algorithms can further minimize the errors in the stability evaluation of stable reference genes^23,24,44,45^. Therefore, a comprehensive evaluation of the results of all four algorithms was also carried out using RefFinder to select the most suitable reference genes for RT-qPCR studies. The ranking of candidate reference genes using RefFinder showed the data was almost in agreement with that of NormFinder and ΔCt method and thereby paving the way to use these genes as the stable reference genes for the accurate normalization of target gene expression in barnyard millet under abiotic stress conditions. Our findings are in accordance with several reports where the comprehensive results obtained from the RefFinder are consistent with the results of geNorm, NormFinder, and RefFinder under different abiotic stress conditions in crop plants^24,46^. In addition, the geNorm program pairwise variation analysis was done to determine the optimal number of reference genes required for normalization^26^. Since the inclusion of more than one reference gene would help in the precise normalization of gene transcript for RT-qPCR analysis^47^. The result of pairwise variation analysis of all samples in this study revealed that the V2/3 value was below the threshold of 0.15. The lower pairwise cutoff range is not surprising because the value of pairwise variation was below the threshold level and similar trend was also reported across various abiotic stress conditions in foxtail millet^13^, pearl millet^48^, and fescue grass^49^. Therefore, using either single or in a combination of top two reference genes would guarantee an accurate result in gene expression studies under abiotic stress conditions.

To further verify the accuracy and reliability of the results normalized by RefFinder, the expression level of *SOD1* was evaluated under various abiotic conditions using selected candidate reference genes (*ɑ-TUB*, *APRT*, *EF-1ɑ*, *GAPDH*, *RP* II, *UBC5* & *ß-TUB*) and their combinations. *SOD*, the front-line reactive oxygen species (ROS) scavenging enzyme, initiates the defense system by removing superoxide accumulated during various abiotic and biotic stressors^50^. Based on their metal cofactors, *SOD* is classified into three distinct groups *Cu/ZnSOD*, *MnSOD*, and *FeSOD*^33^. The enhanced or increased expression of *Cu/Zn-SOD* (*SOD1*) has been reported in plants that possess resistance against drought, salinity, cold, heat, and water-logging stresses in Arabidopsis^51^, tomato^52^, rice^53,54^, wheat^55^, cotton^56^, tea^57,58^, barely^34^ and brassica^59^.

*ɑ-TUB*, an important cell structural protein has been verified to be stably expressed in plant species^16,22,60^, and the present study of *ɑ-TUB*, showed wide expression stability along with *UBC5*, another important gene for ubiquitination cycles that involves a series of enzyme catalytic effects under various environmental conditions. Moreover, *ɑ-TUB* and *UBC* were reported to be the most stable reference genes under drought, salinity, and cold stresses in pearl millet^48^, golden spider lily^32^ and jute^61^. *GAPDH* an abundant glycolytic enzyme has also been extensively used as a reference gene in many RT-qPCR experiments in many plant species. In the present study, it has stable expression in both salinity and cold stress conditions when used alone or in combination with *APRT*, a key enzyme involved in the purine salvage pathway. Several RT-qPCR studies in crop plants suggested *GAPDH* as the most stable gene that can be used as reference under drought^38^ and salinity^62^, and *APRT* in cold experimental conditions^63^. *EF-1ɑ* has also been reported as the most stable reference gene in finger millet^64^, pearl millet^48^, grass pea^62^ and wheat^65^ under different combinations of abiotic stresses, and in the present study, *EF-1ɑ* along with *RP* II (cellular translation proteins) alone or in combination showed stable expression in heat stress conditions. The stability of reference genes strongly influences the accuracy of RT-qPCR results^62^. When we used the most stable genes, to analyze the RT-qPCR data, the expression results matched the trend observed in the *SOD*1 activity. Contrastingly, when the least stable reference genes, *TLF* & *β-TUB* were used for normalization of the RT-qPCR data produced altered trends of the expression patterns (over or underestimated), i.e., inconsistent with the experimentally measured *SOD*1 activity. These findings demonstrate that a more stable reference gene increases the accuracy of the results. The results are in agreement with those data reported in sorghum^66^ and garlic^37^, which demonstrated that normalization using unstable reference genes can lead to faulty RT-qPCR results.

## Conclusion

In the edaphic climatic scenario, the orphan minor millets research has been directed towards understanding the molecular mechanisms of plant responses to abiotic stresses and their combinations through functional genomics approaches, particularly transcriptomics. However, the accuracy or reliability of transcriptomics data or gene expression data through RT-qPCR strongly depends on the normalization of a gene of interest using suitable reference genes. This study represents the first attempt to select a candidate reference genes in barnyard millet under drought, salinity, cold and hot stress for the normalization of gene expression data using RT-qPCR. The RT-qPCR results were analyzed using five different algorithms. We identified *UBC5* and *ɑ-TUB* as the most stable reference genes for drought stress, and *ɑ-TUB* and *GAPDH* as the most stable for salinity conditions. *GAPDH* is the best, and *APRT* is the second-best reference gene for cold treatment, while *EF-1ɑ* and *RP* II are the most stable genes under heat stress. For all treatments, the unstable gene was *ß-TUB*. Moreover, the *SOD1* expression study under abiotic stress conditions has laid the foundation for in-depth research in barnyard millet due to the significant changes in their expression after stress treatment. The identified reference genes will not only enable reliable gene expression studies but also would be helpful in functional genomics and metabolomics of abiotic stress tolerance in barnyard millet.

## Materials and methods

### Plant materials and stress treatments

Seeds of MDU 1 variety of barnyard millet (*Echinochloa colona* var *frumentacea*) were collected from the Department of Plant Breeding and Genetics, Agricultural College and Research Institute, Tamil Nadu Agricultural University, Madurai. Seeds of MDU 1 were raised under hydroponics conditions with a complete Yoshida nutrient medium under a greenhouse environment^67^. The greenhouse condition was maintained with a relative humidity range of 60–70%, a photoperiod of 12:12 h, and a temperature range of 22 ± 2 °C (night) and 26 ± 2 °C (day). Twenty-one-day-old seedlings were then subjected to four different abiotic stressors viz., drought, salinity, cold and heat as followed in foxtail millet^13^. For drought stress, the roots of the seedlings were placed in a conical flask containing 20% polyethylene glycol (PEG 6000). For salinity treatment, the roots of the seedlings were dipped in conical flask containing 250 mM NaCl solution. For cold and heat treatments, the seedlings were kept at 4 °C (refrigerator) and 41 °C (incubator), respectively. Leaf samples were collected after the seedlings were exposed to 0, 12, 24, 36, and 48 h of various stress induction^13^. Untreated seedlings were maintained as controls. Two independent biological replicates were maintained for each of the samples. The collected samples were immediately flash-frozen in liquid nitrogen and kept at − 80 °C for subsequent use.

### RNA isolation and Reverse Transcription

Total RNA was extracted from leaf samples of respective treatments using RNAiso Plus (TaKaRa Bio, India) following the manufacturer’s guidelines. RNA was then purified using RNase-free rDNase (NucleoSpin, MACHEREY–NAGEL) according to the manufacturer’s protocol. The quality and quantity [A260/280 & A230/280] of the RNA samples were determined using a Genova plus Life Science Spectrophotometer (JENWAY, UK). The RNA samples with A260/A280 ratio between 1.9 and 2.1 and A260/A230 ratio greater than 2.0 were considered for cDNA conversion. The RNA integrity was also assessed by agarose gel electrophoresis (1.5%).

Reverse transcription PCR (cDNA synthesis) was performed with PrimeScript™ RT reagent Kit (TaKaRa Bio India) following the manufacturer’s protocols. Priming reactions were carried out in a total final volume of 10 µl reaction containing total RNA (5 µg), oligo (dt) primers (1 µl), dNTPs (1 µl), and RNase free water (variable). The prepared mixture was kept for 5 min at 65 °C (priming); cooled immediately on ice; reverse transcription was done in a total volume of 20 µl reaction containing primed 10 µl RNA (5 µg), 4.0 µl buffer (5X), 0.5 µl RNase inhibitor (20 U), 1.0 µl RT enzyme (200 U/µl) and RNase free water (variable). The reaction mixture was mixed thoroughly and kept for 30 min at 42 °C; finally, the inactivation of the enzymes was done for 15 min at 70 °C and the obtained cDNA was stored at -20 °C.

### Selection and design of reference and target genes for quantitative real-time PCR

A total of ten candidate references and one target gene were selected based on the literature on other crop plants. Candidate reference genes include *Actin* (*ACT*), α-*tubulin* (*α-TUB*), *β-TUBulin* (*β-TUB*), *RNA pol* II (*RP* II), *elongation factor-1 alpha* (*EF-1α*), *adeninephosphoribosyltransferase* (*APRT*), TATA-*binding protein-like factor* (*TLF*), *ubiquitin-conjugating enzyme* 2 (*UBC2*), *ubiquitin-conjugating enzyme* E2 L5 (*UBC5*) and *glyceraldehyde*-3-*phosphate dehydrogenase* (*GAPDH*), which were the common house-keeping genes in crop and animal species. *Cu/Zn-superoxide dismutase* [*SOD* (*Cu–Zn*)] or *SOD1*, an important antioxidant enzyme that expressed during tolerance against various abiotic stressors in plant species was selected for reference gene validation^68^. The CDS (coding sequence) of these genes were retrieved from *E. crus-galli* (wild) and *E. colona* var *frumentacea* (cultivated) which were available in the National Center for Biotechnology Information (NCBI) and National Genomics Data Center (NGDC) database, respectively, for RT-qPCR experiments. Then the respective gene CDS was searched for sequence homologs using BLASTn. The top E-score (< 0.0) with the greatest similarity and organism identity (plants of cereal family) of five for respective genes were selected for multiple sequence alignment (CLC genomics workbench) to identify conserved regions and for primer designing. Finally, the primers were designed [PrimerQuest™ Tool-Integrated DNA Technologies (IDT)] with the following criteria to meet RT-qPCR requirements, including melting temperature of around 60 °C; GC content of 40–60%; primer length of 20–22 bp; and amplicon size of 90 to 200 bp. A primer pair spanned the exon-exon junction was selected for RT-qPCR study to avoid amplification of gDNA in possibly contaminated samples. The designed primers were analyzed for limited/absence of self- and hetero-dimer using the OligoAnalyzer tool (IDT). All the primers were synthesized from Sigma Aldrich, USA (Supplementary Table S2).

### Quantitative real-time PCR conditions

Quantitative real-time PCR (RT-qPCR) was performed and analyzed using the CFX96 Touch Real-Time PCR Detection System (Bio-Rad) and accompanying CFX96 Maestro Software (Bio-Rad). Each RT-qPCR reaction contained 50 ng of cDNA (1μL), 2 nmoles each of forward (1μL) and reverse primer (1μL), and 5μL of TB Green Premix Ex Taq (Tli RNase H Plus) and water added to make up the total volume of 10μL. The RT-qPCR thermal conditions were as follows; initial denaturation at 95 °C for 30 s, followed by 39 cycles of denaturation at 95 °C for five seconds and primer annealing and extension were at 60 °C for 30 s. Melt-curve analyses was performed to test the primer specificity by heating from 65 to 95 °C with a stepwise increase of 0.5 °C every 10 s. In addition, each PCR reaction included a non-RT (without cDNA) and non-TB green (without SYBR) control to check the contamination in genomic DNA and reagents mix respectively.

### Evaluation of primer’s specificity and amplification efficiency

The melt-curve cycle with a single peak in RT-qPCR and a single band of the expected size in 1% agarose gel electrophoresis was used as criteria to ensure the specificity of amplification (primers) for each reference gene. The amplification efficiency for each gene primer was determined by performing RT-qPCR using tenfold serially diluted cDNA of the control plant. The standard curve values (Cq; quantification cycle) obtained from a serial dilution revealed the amplification efficiency (E) and correlation coefficient (R^2^) of respective gene primers using the formula, E = [10^(1/-slope)^ − 1] × 100%^69^. Two biological and technical replicates were performed in all RT-qPCR.

### Software and data analysis

The average Cq values calculated from RT-qPCR data for all samples or genes were used for further stable gene identification. Four excel based statistical software namely geNorm^26^, NormFinder^27^, BestKeeper^28^, ΔCt^29^, and one web-based algorithm RefFinder^30^, were applied to compare the expression stability of the reference genes across all treatments. For geNorm, M value (expression stability value) and V_n_/V_n+1_ (pairwise variation) values were calculated for all candidate genes. Where, the lower the M value higher the stability of the gene and *vice-versa*. Similarly, if any primers with V_n_/V_n+1_ value is lower than the cut-off value, the normalization requires additional reference gene and *vice-versa*. NormFinder measures the stability value of each tested reference gene according to inter-group and intra-group variations. The lower the values of intra- and inter-group variations, the greater the stability of the gene and *vice-versa*. The BestKeeper method determines the stability value of a gene based on the standard deviation (SD) and coefficient of variations (CV) of Cq values. The most stable gene expression exhibits the lowest CV ± SD value. RefFinder program was used to obtain a comprehensive ranking based on the geometric mean of all the candidate reference genes from all three methods mentioned above. For validation of selected reference genes as ideal, the 2^(−Delta Delta Ct) or 2^–∆∆Ct^ method was applied to calculate the relative expression level of selected reference and target gene^70^.

### Ethical approval

We state that this research was not carried out with human or any animal subjects.

### Supplementary Information

Supplementary Information.

## Data Availability

The primer sequences used in this study are included in Supplementary Table S2. And all the CDS sequence data and Cq values of each treatment are also included in Supplementary Tables S1 and S3 respectively.

## Material availability

The seeds of MDU 1 barnyard millet were collected from the Department of Plant Breeding and Genetics, Agricultural College and Research Institute, Madurai, Tamil Nadu, India. The plant materials used in this study complies with the IUCN Policy Statement on Research Involving Species at Risk of Extinction and the Convention on the Trade in Endangered Species of Wild Fauna and Flora policies. We also state that MDU 1 is a cultivated variety in Tamil Nadu and it is neither a protected nor patented variety to propagate or sell.
