# Supplementary material for: Selection and validation of reliable reference genes for quantitative real-time PCR in Barnyard millet (Echinochloa spp.) under varied abiotic stress conditions
Source: Sci Rep. 2023 Sep 20;13:15573. doi: 10.1038/s41598-023-40526-6 (PMC10511452; doi:10.1038/s41598-023-40526-6)
Supplement: Supplementary file 1 — Supplementary Information. [file 41598_2023_40526_MOESM1_ESM.pdf]

Selection and validation of reliable reference genes for quantitative real-time PCR in Barnyard millet (*Echinochloa* spp.) under varied abiotic stress conditions

Vellaichamy Gandhimeyyan Renganathan[1‡], Raman Renuka[1*‡], Chockalingam Vanniarajan[2], Muthurajan Raveendran[3], and Allimuthu Elangovan[3]

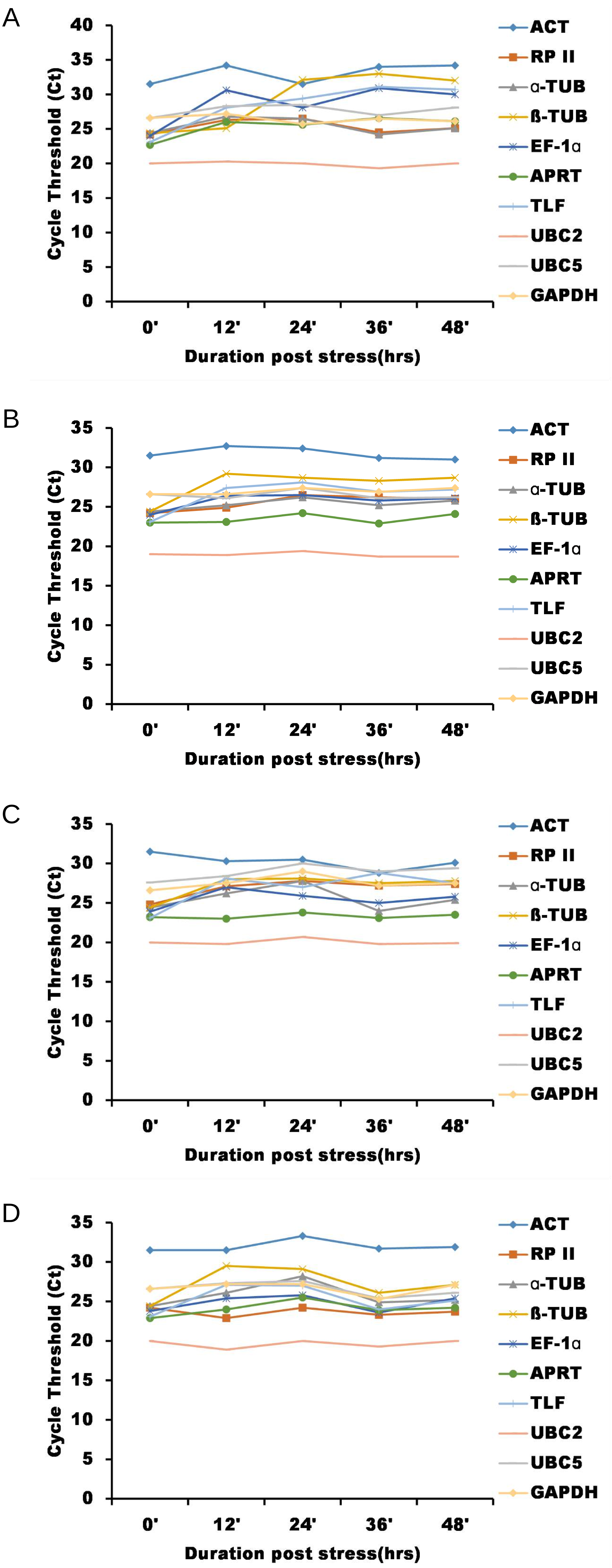

Supplementary Fig. s1. The mean Ct/Cq values of 10 candidate reference tested under (A) Drought, (B) Salinity, (C) Cold, and (D) Hot conditions at different time points.

Supplementary Table s1. Details of CDS sequences of *Echinochloa* spp. used for designing qRT-PCR primers

S. No	Gene Name	Location	Sequence/source
1	*ACT*	scaffold50.371	ATGGCTGACGGCGAAGACATCCAGCCGCTTGTGTGTGACAATGGGACCGGCATGGTCA<br>AGGCTGGTTTTGCTGGTGATGATGCACCAAGGGCTGTTTTCCCAAGTATTGTTGGGCGT<br>CCCCGTCACACTGGTGTGATGGTTGGCATGGGGCAGAAGGATGCGTATGTTGGTGATG<br>AGGCCCAGTCCAAGAGGGGTATCCTGACCTTGAAGTACCCGATCGAGCATGGTATTGTT<br>AGCAACTGGGATGATATGGAGAAGATCTGGCACCACACCTTCTACAATGAACTACGTG<br>TTGCACCTGAGGAGCACCCTGTGCTGCTGACTGAAGCCCCACTCAACCCCAAGGCTAAC<br>AGGGAGAAGATGACCCAGATCATGTTTGAGACCTTCAATGTTCCTGCCATGTATGTTGC<br>CATCCAGGCTGTGCTTTCCTTGTATGCCAGTGGACGGACAACTGGTATCGTTTTGGACT<br>CTGGTGATGGTGTCAGCCACACTGTGCCAATCTATGAAGGTTATGCCCTTCCTCATGCC<br>ATCCTCCGTCTTGACCTTGCTGGGCGTGACCTGACTGACAGCCTGATGAAGATTCTCAC<br>TGAGAGAGGTTACTCCTTCACCACCACTGCTGAACGGGAAATCGTAAGGGACATCAAG<br>GAAAAGCTCGCGTATGTGGCTCTTGACTACGAGCAGGAGCTGGAGAATGCGAAGACCA<br>GCTCATCTGTGGAGAAAAGCTACGAGCTGCCTGATGGTCAGGTGATCACCATTGGGGC<br>AGAGAGGTTTAGATGCCCTGAGGTCCTCTTCCAGCCTTCCATCATTGGTATGGAAGCTC<br>CTGGCATCCATGAGACCACCTACAACTCCATTATGAAGTGTGATGTGGATATTAGGAAG<br>GACTTGTATGGTAACATTGTGCTCAGTGGTGGCACGACCATGTTCCCTGGTATCGCGGA<br>CCGTATGAGCAAGGAGATCACTGCCCTTGCACCGAGCAGCATGAAAATTAAGGTGGTG<br>GCACCACCTGAAAGGAAATACAGTGTCTGGATAGGAGGGTCCATCCTTGCCTCACTTA<br>GCACCTTCCAACAGATGTGGATATCAAAGGCGGAGTACGATGAGTCGGGGCCAGCAAT<br>TGTTCACCGGAAGTGCTTCTAA
2	*RP II*	scaffold377.22	ATGGACCGTCCGGCGGCCGGCGCGTCGTACCAGCGCTTCCCCCGCGTGAAGATCCGCG<br>ACCTCAAGGACGACTTCGCCAAGTTCGAGCTCCGCGACACCGACGCCAGCATGGCCAA<br>CGCCCTCCGCCGCGTCATGATCGCGGAGGTCCCCACCGTCGCCATCGACCTCGTCGAGA<br>TCGAGGTCAACTCCTCCGTCCTCAACGACGAGTTCATCGCGCACCGCCTGGGGCTCATC<br>CCCCTCACCTCCTCCGCCGCCATGCAAATGCGATTCTCCCGGGACTGCGACGCCTGCGA<br>CGGTGACGGCTCCTGCGAGTTCTGCTCCGTCGAGTTCAACCTCCACGCGCGCGCCGAAT<br>CCGACCAGACGCTCGAGGTCACATCCAACGACCTGAGGTCCTTGGACCCCAAGGTATG<br>CCCCGTCGACCAGGCACGCGCCTACCAGCAGGCGCTCGGTGGCGCTGACGGCTTTGAC<br>CCCAACGAGCAGAGGCAAGTCATCCTTCATTTACACGATAGGGATGAAGAAGCATGGG<br>GAATACTTATTGTAAAGCTGCGTCGGGGGCAAGAACTGAGGCTTCGTGCCATTGCTAG<br>GAAGGGAATCGGCAAGGACCATGCCAAATGGTCACCAGCTGCTACCGTGACTTTCATG<br>TATGAGCCTGACATACGTATCAATGAAGAACTCATGGATACATTGACGCTTGATGAGA

			AAAGAAGCTGGATCGAAAGCAGCCCTACAAAAGTATTTGAGCTTGATAATGTTACCCA<br>GCAGGTGACGGTGGTTGACAATGGTGAGGCATACACCTATGACGATGAGGTGATCAAG<br>AAGGCGGAGGCCATGGGAAAGCCGGGCTTAGTGGAGATCAATGCGAAGGAGGACAGC<br>TTCATCTTCACCGTGGAAACGACGGGCGCCATCACGGCCTACGAGCTGATCATGAACG<br>CCATCACGGTCCTGAGGCAGAAGCTGGATGCCGTCCGCCTCCAGGACGACGACAGCGA<br>CCTTGGCGAGCTCGGCGCCCATCTCGGTGGA
3	*a-TUB*	scaffold2.684	ATGAGAGAGATCATCAGCATCCACATCGGCCAGGCCGGGATCCAGGTCGGCAACTCCT<br>GCTGGGAGCTCTACTGCCTCGAGCACGGCATCGAGCCCGATGGCACCATGCCAAGTGA<br>TACCTCGGTTGGCGTCGCACATGATGCCTTCAACACCTTCTTCAGTGAGACCGGCTCTG<br>GCAAGCATGTGCCGAGGGCCATCTTTGTTGACCTTGAGCCCACTGTCATCGATGAGGTG<br>CGCACTGGCTCATACCGCCAGCTCTTCCACCCAGAGCAGCTCATCTCTGGGAAGGAGG<br>ATGCGGCTAACAACTTTGCCCGTGGCCACTACACTGTTGGAAAGGAGATTGTCGATCTC<br>TGCCTGGACCGCGTGCGCAAGCTAGCAGACAATTGCACTGGGCTGCAGGGATTCTTGG<br>TGTTCAATGCTGTTGGTGGTGGTACTGGCTCTGGACTTGGTTCACTGCTGTTGGAGCGC<br>CTCTCAGTTGATTACGGCAAGAAGTCTAAGCTCGGCTTCACCATTTATCCGTCCCCACA<br>GGTGTCAACAGCTGTTGTAGAGCCATACAACAGTGTCCTCTCTACCCACTCCCTGCTTG<br>AGCACACAGATGTTGCAGTCCTCCTGGACAATGAGGCAATCTATGACATATGCCGGAG<br>GTCCCTTGACATTGAAAGGCCAACCTACACCAACTTGAACAGGCTGATCTCACAGATCA<br>TATCTTCGCTTACCACCTCCCTGAGATTTGATGGTGCTATCAACGTGGATGTTACAGAG<br>TTCCAGACCAACCTTGTTCCATACCCACGCATCCATTTCATGCTTTCATCATATGCCCCT<br>GTAATCTCTGCCGAGAAGGCCTACCATGAGCAGCTCTCTGTGCCCGAAATCACCAATGC<br>TGTCTTTGAGCCCTCAAGCATGATGGCCAAGTGTGACCCAAGGCATGGGAAGTACATG<br>GCTTGCTGCTTGATGTACCGTGGTGATGTTGTTCCCAAGGATGTCAATGCTGCCGTTGC<br>AACCATCAAGACCAAGAGAACTGTCCAGTTTGTGGACTGGTGCCCCACTGGATTCAAG<br>TGTGGCATCAACTACCAGCCACCCTCTGTTGTCCCTGGAGGCGACCTGGCAAAGGTCCA<br>GCGTGCCGTGTGCATGATCAGCAACAACACAGCCGTTGCTGAGGTGTTCTCGCGCATCG<br>ACCACAAGTTCGACCTGATGTATGCTAAGCGTGCCTTCGTGCACTGGTACGTTGGTGAG<br>GGTATGGAGGAAGGCGAGTTCTCAGAGGCCCGTGAGGACTTGGCTGCCCTTGAGAAGG<br>ACTACGAGGAGGTTGGCGCAGAGGGCGCAGACGACGAAGGTGACGAGGGAGAAGACT<br>ATTGA

4	*ß-TUB*	scaffold3.941	ATGAGGGAGATCCTACACATCCAGGGCGGGCAGTGCGGGAACCAGATCGGCGCCAAGT TCTGGGAGGTGATCTGCGACGAGCACGGCATCGACGCCACGGGCCGCTACGCGGGGGA CTCGGACCTCCAGCTCGAGCGCATCAACGTCTACTACAACGAGGCCAGCGGGGGCCGG TTCGTGCCGCGCGCCGTGCTCATGGACCTCGAGCCCGGGACCATGGACTCCGTCCGCTC CGGCCCCTTCGGCCAGATCTTCCGCCCCGACAACTTCGTCTTCGGCCAGTCCGGCGCCG GCAACAACTGGGCCAAGGGACACTACACCGAGGGCGCCGAGCTCATCGACTCCGTCCT CGACGTCGTCCGCAAGGAGGCCGAGAACTGCGACTGCCTCCAGGGTTTCCAAGTTTGC CATTCCTTGGGAGGAGGCACTGGTTCTGGCATGGGCACCCTTCTTATTTCTAAGATCAG GGAGGAGTACCCTGATAGAATGATGTTGACCTTCTCCGTATTCCCATCACCAAAGGTTT CAGATACTGTTGTGGAGCCATACAATGCTACACTTTCGGTTCACCAACTTGTTGAGAAT GCTGATGAATGTATGGTGCTTGACAATGAAGCTCTGTATGACATCTGCTTCCGCACACT AAAGCTTGCCACCCCTACCTTTGGTGACCTGAACCATCTTATCTCTGCTACCATGAGTG GCGTCACGTGCTGCCTGAGGTTCCCTGGTCAGCTCAACTCCGACCTGCGCAAGCTTGCC GTGAACCTCATCCCATTCCCTCGTCTCCATTTCTTCATGGTCGGCTTTGCGCCGCTGACC TCACGTGGATCGCAGCAGTACCGCGCCCTCACTGTCCCTGAGCTGACCCAGCAGATGTG GGATGCCAAGAACATGATGTGTGCCGCTGACCCCCGCCACGGCCGTTACCTCACGGCCT CCGCCATGTTCCGTGGGAAGATGAGCACCAAGGAGGTGGACGAGCAGATGCTCAACGT CCAGAACAAGAACTCGTCTTACTTCGTGGAGTGGATCCCGAACAATGTCAAGTCCAGT GTGTGCGACATCCCACCCAAGGGTCTGAAGATGGCGGGTACCTTCGTCGGGAACTCCA CCTCCATTCAGGAGATGTTCCGCAGGGTGAGCGAGCAGTTCACGGCCATGTTCAGGCG CAAGGCCTTCTTGCACTGGTACACCGGCGAGGGGATGGACGAGATGGAGTTCACCGAA GCTGAGAGCAACATGAATGACCTGGTTGCGGAGTACCAGCAGTACCAGGACGCCACCG CCGACGAGGAGTATGAGGACGAGGAGGAAGAGGAGGAGGCGGCCGAATAA
5	*EF-1a*	scaffold8.559	ATGGGTAAGGAGAAGTCCCACATCAACATCGTGGTCATTGGCCACGTCGACTCTGGCA AGTCGACCACCACTGGCCACCTGATCTACAAGCTTGGAGGTATTGACAAGCGTGTGATC GAGAGGTTCGAGAAGGAGGCTGCTGAGATGAACAAGCGGTCCTTCAAATACGCGTGGG TGCTTGACAAGCTCAAGGCTGAGCGTGAGAGAGGTATCACCATTGATATCGCCCTGTG GAAGTTCGAGACCACCAAGTACTACTGCACTGTGATTGACGCCCCTGGACACCGTGACT TCATCAAGAACATGATCACTGGTACCTCCCAGGCTGACTGTGCTGTCCTTATCATTGAC TCCACCACTGGTGGTTTTGAGGCTGGTATCTCCAAGG
6	*APRT*	scaffold29.598	ATGCTGCCCCCCCTGCGGTTCCAGCCTCGCCTCGGCCTCGGCCTCCCGTCGTCAGCAGC AGCAGCAGCGTCGGCGCCGCCGCCGCCTTTTCCTCCGCGCGGGCCCGCCGCCACCACCA CCGGCGTCTCCGTCGGCGGCGCGAGACCCAGCGCGCGTGTCACCGGCGGGAGGCGGCG AGGGCCGGCGGTGGCGATGGCGTCCGGCGACGCGCGCGTGGCGGGGATCGCGTCCTCC ATCCGCGTCATCCCCGACTTCCCCAAGCCAGGAATCATGTTTCAGGATATCACGACATT GCTCCTCGATCCGAAGGCGTTCCGTGACACGATCGACCTCTTTGTTGAGCGCTACAAGG

			ACCAAGGGATCACCGTAGTTGCTGGTGTTGAAGCTAGGGGTTTTATCTTTGGCCCTCCT ATTGCTTTAGCCATAGGTGCAAAATTTGTACCTTTGAGAAAGCCAAAAAAGTTGCCAGG CGAGGTGATCTCTGAAGAATATTCTTTGGAATATGGCACCGACAAAATAGAAATGCAT GTTGGAGCTGTGGAGGCCAATGATCGAGCACTTGTTGTCGATGATCTCATTGCCACTGG TGGAACACTATGTGCAGCTGTCAAACTTCTCGAGCGTGTTGGAGCAAAGGTGGTTGAGT GTGCTTGTGTCATTGAATTGCCAGAATTGAAGGGCCGGGACAAGCTAGGGGACAGGCC CGTTTTCGTCCTTGTG
7	*TLF*	scaffold89.132	ATGGTGAAGTTCACGGCGGAAGAGCTCCGTGGCATCATGGACAAAAAGAACAACATTC GTAATATGTCCGTTATTGCTCATGTGGACCATGGCAAGTCTACCCTTACAGATTCCCTTG TGGCAGCTGCTGGTATCATTGCCCAGGAAGTTGCTGGTGATGTTCGCATGACTGATACT CGTGCAGATGAAGCAGAGCGTGGTATTACAATCAAATCCACTGGTATATCTCTTTACTA TGAGATGACTGAGGAGTCACTGAAGAATTACAAGGGTGAGAGAGATGGTAGCGAATA CTTGATCAACCTTATTGACTCACCTGGGCACGTTGATTTTTCTTCGGAAGTCACAGCTGC TCTTCGTATCACCGATGGTGCTCTAGTTGTGGTTGACTGTATTGAAGGTGTCTGTGTGCA AACTGAAACTGTGCTCCGCCAGGCTCTTGGTGAGAGGATTAGGCCAGTCCTTACTGTGA ACAAGATGGACAGGTGCTTCCTTGAGCTTCAGGTTGAGGGTGAGGAAGCCTACCAGAC TTTCTCCCGTGTCATTGAGAATGCCAATGTCATTATGGCTACATATGAAGATAAGCTCC TAGGTGATGTCCAAGTCTACCCAGAGAAGGGAACTGTTGCTTTCTCTGCTGGTCTGCAT GGCTGGGCCTTCACCCTCACCAACTTTGCCAAGATGTATGCATCCAAGTTTGGAGTTGA TGAATCTAAGATGATGGAGAGGCTCTGGGGTGAGAACTTTTTTGACCCAGCCACAAAG AAGTGGACCACGAAGAACACAGGCTCTGCTACCTGCAAGAGAGGATTTGTTCAGTTCT GCTATGAGCCAATCAAGCAAATCATCAACACCTGCATGAATGACCAGAAGGATAAGTT GTGGCCCATGCTTCAAAAGCTTAATGTTACCATGAAGGCTGATGAGAAGGAATTGGTT GGCAAGGCTTTGATGAAGCGTGTTATGCAAACCTGGCTCCCAGCCAGTACTGCCCTACT TGAGATGATGATATTCCACCTCCCTTCCCCATCAAAGGCACAAAAGTATCGTGTGGAGA ACTTGTACGAGGGACCCCTTGATGATATCTATGCTACTGCTATCAGGAACTGTGATCCA GAGGGTCCTCTTATGCTGTATGTTTCAAAGATGATTCCAGCATCTGACAAAGGCAGGTT CTTTGCCTTCGGTCGTGTCTTCTCAGGGAAGGTCGCTACTGGTATGAAGGTTCGAATCA TGGGTCCCAACTATGTCCCTGGCCAGAAGAAGGATCTATATGTCAAGAGTGTCCAGCGT ACTGTTATCTGGATGGGAAAGAAACAAGAGTCAGTTGAGGATGTTCCTTGTGGTAACA CTGTTGCTATGGTTGGTCTGGATCAGTTCATCACAAAGAATGCTACACTCACTAATGAG AAGGAGGTTGATGCATGCCCAATCAGAGCAATGAAGTTCTCTGTCTCCCCTGTTGTGCG TGTTGCTGTTCAGTGCAAGGTTGCCTCTGACCTACCCAAGCTAGTTGAAGGTTTGAAGC GTCTCGCAAAGTCTGATCCTATGGTCCTCTGTACAATTGAAGAATCTGGTGAGCATATT ATTGCTGGAGCTGGTGAGCTTCATCTGGAGATTTGCCTGAAGGATCTGCAGGAAGACTT CATGGGTGGTGCTGAGATTATTGTTTCCCCGCCTGTTGTCTCTTTCCGTGAAACTGTTCT

			TGAGAAATCCTGCCGAACAGTCATGAGTAAGTCCCCCAACAAGCACAACCGTCTTTAC<br>ATGGAAGCCCGCCCCTTGGAAGAGGGTCTTGCTGAGGCCATTGATGAAGGTCGCATTG<br>GTCCACGTGATGATCCCAAGGTGCGCTCCAAGATCCTCTCTGAGGAGTTCGGCTGGGAC<br>AAGGATCTTGCCAAGAAGATTTGGTGCTTTGGACCTGAGACCACCGGCCCGAACATGG<br>TTGTTGATATGTGTAAGGGAGTGCAGTACCTCAATGAAATCAAGGATTCTGTGGTGGCT<br>GGTTTCCAGTGGGCCTCAAAGGAGGGTGCGCTAGCTGAGGAGAACATGCGTGGAATTT<br>GCTTTGAGGTCTGTGATGTTGTTCTTCACGCTGATGCAATTCACAGGGGTGGTGGCCAG<br>GTCATTCCAACTGCCAGGAGGGTCATTTATGCTTCTCAGCTGACGGCCAAGCCAAGGCT<br>GCTCGAGCCAGTTTACCTTGTGGAGATCCAGGCCCCAGAAAATGCACTTGGTGGTATCT<br>ATGGTGTTCTGAACCAGAAGAGAGGGCACGTGTTTGAGGAGATGCAGAGGCCGGGGAC<br>CCCGCTCTACAACATCAAGGCTTACCTCCCTGTCATCGAGTCATTTGGGTTCTCAAGCC<br>AATTGAGGGCTGCAACCTCTGGCCAGGCGTTCCCCCAGTGTGTGTTTGACCACTGGGAC<br>ATGATGGGCTCTGATCCTCTGGAGGCTGGCTCCCAGGCCGCTCAGCTGGTGTTGGATAT<br>CCGCAAGAGGAAGGGTCTCAAGGAACAGATGACCCCTCTTTCTGAGTTCGAGGACAAG<br>CTCTAA
8	*UBC2*	*E.frumentacea* mRNA GWHTBDNQ043355	https://ngdc.cncb.ac.cn/gwh/Assembly/21453/show
9	*UBC5*	*E.frumentacea* mRNA GWHTBDNQ048013	https://ngdc.cncb.ac.cn/gwh/Assembly/21453/show
10	*GAPDH*	*E.frumentacea* mRNA GWHTBDNQ085699	https://ngdc.cncb.ac.cn/gwh/Assembly/21453/show
11	*SOD1*	*E.frumentacea* mRNA GWHTBDNQ090839	https://ngdc.cncb.ac.cn/gwh/Assembly/21453/show

Supplementary Table s2. Details of primer sequences and amplicon characteristics of candidate reference and target genes

S. No	Gene	Genome location	Primer sequences (5'3') Forward/Reverse	Amplicon size (bp)	Tm (°C)	E (%)	R^2
**Reference genes**							
1	*Actin* (*ACT*)	*E.crus-galli* Scaffold50.371	**F:** CAAGGCTAACAGGGAGAAGATG<br>**R:** TCCACTGGCATACAAGGAAAG	120	62	93.7	0.97
2	*RNA polymerase II* (*RP* II)	*E.crus-galli* mRNA	**F:** TGTTGTGAGGAAGGAAGCTG<br>**R:** GTGGCATTGTATGGCTCAAC	110	62	95.4	0.98
3	*Tubulin- a* (*a-TUB*)	*E.frumentacea* mRNA Scaffold2.684	**F:** CCATACAACAGTGTCCTCTCTAC<br>**R:** CCTCCGGCATATGTCATAGATT	96	62	110.1	0.97
4	*Tubulin-ß* (*ß-TUB*)	*E.crus-galli* Scaffold3.941	**F:** TCCGTATTCCCATCACCAAAG<br>**R:** GCTTCATTGTCAAGCACCATAC	116	62	102.4	0.99
5	*Elongation factor -1a* (*EF-1a*)	*E.crus-galli* Scaffold8.559	**F:** AGGCCCGTTATGATGAGATTG<br>**R:** AAACCAGAGATTGGGACGAAG	97	62	102.1	0.99
6	*Adenine phosphoribosyltransferase* (*APRT*)	*E.crus-galli* Scaffold29.598	**F:** TGGTGGAACACTATGTGCAG<br>**R:** GCCCTTCAATTCTGGCAATTC	97	62	100.7	0.98
7	*TATA-Binding Protein (TBP)-Like Factor* (*TLF*)	*E.crus-galli* Scaffold89.132	**F:** GCTACCTGCAAGAGAGGATTT<br>**R:** AGCATGGGCCACAACTTAT	101	62	105.6	0.99
8	*Ubiquitin-conjugating enzyme 2* (*UBC2*)	*E.frumentacea* mRNA *GWHTBDNQ043355*	**F:** CTTCTATTCAGTCCCTGCTGTG<br>**R:** GTTGTACTCCCGCTTGTTCT	95	62	99.4	0.99
9	*Ubiquitin-conjugating enzyme* E2 5 (*UBC5*)	*E.frumentacea* mRNA *GWHTBDNQ048013*	**F:** CCTTGCACAAATACCTCGTAATG<br>**R:** GCGGAGAGTCCTGATTTCTTAG	101	62	105.2	0.98
10	*Glyceraldehyde-3-phosphate dehydrogenase* (*GAPDH*)	*E.frumentacea* mRNA *GWHTBDNQ085699*	**F:** TGAAGGATTGGAGAGGAGGA<br>**R:** TTTCCGTTCAGCTCAGGTAAG	103	62	91.2	0.99
**Target gene**							
1	*Cu/Zn-binding superoxide dismutase* (*SOD1*)	*E.frumentacea* mRNA *GWHTBDNQ090839*	**F:** GGACCACATTTCAACCCAAAC<br>**R:** AATGGTTGCCTCAGCTACTC	117	62	106.3	0.99

Supplementary Table s3. Cq values of reference gene under time intervals of varied stress treatment

Treatment	Post duration hours	Replicates	*ACT*	*RP II*	*a-TUB*	*ß-TUB*	*EF-1a*	*APRT*	*TLF*	*UBC2*	*UBC5*	*GAPDH*
**PEG 6000**	**0'**	**RI**	31.6	24.2	24.4	24.4	24.0	22.7	23.1	20.0	26.6	26.6
		**RII**	31.5	24.3	24.3	24.6	24.0	23.3	23.0	20.0	26.5	26.7
		**RIII**	31.5	24.2	25.0	24.2	23.8	22.8	23.1	20.1	26.6	26.4
	**12'**	**RI**	34.2	26.3	26.8	25.1	30.6	26.0	28.1	20.3	28.3	27.2
		**RII**	34.2	26.5	26.0	26.3	30.8	26.1	28.1	20.8	28.7	27.1
		**RIII**	34.2	26.0	26.2	26.6	30.4	25.8	28.4	20.1	28.1	27.2
	**24'**	**RI**	31.5	26.5	26.5	32.1	28.1	25.6	29.4	20.0	28.5	25.7
		**RII**	31.4	26.5	26.6	32.0	28.1	25.5	29.1	20.0	28.4	25.6
		**RIII**	31.6	26.6	26.3	32.0	28.1	25.3	29.5	20.0	28.3	25.6
	**36'**	**RI**	34.0	24.5	24.2	33.0	30.9	26.6	32.1	19.3	27.0	26.5
		**RII**	34.4	23.5	23.9	32.9	30.6	26.6	30.5	19.2	27.8	26.5
		**RIII**	33.7	24.1	24.4	32.5	30.6	26.5	31.4	19.3	27.2	26.4
	**48'**	**RI**	34.2	25.1	25.1	32.0	30.0	26.1	30.5	20.0	28.1	26.1
		**RII**	33.8	24.4	24.9	32.2	30.5	26.4	30.1	19.8	28.0	26.7
		**RIII**	34.1	24.5	25.4	33.1	30.8	27.1	31.0	20.1	28.2	26.3
**NaCl 250mM**	**0'**	**RI**	31.5	24.2	24.4	24.4	24.0	23.0	23.1	20.0	26.6	26.6
		**RII**	31.6	24.3	24.5	24.6	23.9	22.8	23.0	20.0	26.8	26.7
		**RIII**	31.4	24.2	24.4	26.5	24.2	23.7	23.0	20.0	26.4	26.4
	**12'**	**RI**	32.7	24.9	25.2	29.2	26.4	25.1	27.4	18.9	26.1	26.6
		**RII**	32.4	25.1	25.2	29.4	26.6	25.2	27.9	19.2	26.4	26.5
		**RIII**	33.2	24.8	25.3	28.7	26.1	25	26.9	19.5	26.3	26.7
	**24'**	**RI**	32.4	26.5	26.2	28.7	26.5	24.8	28.1	19.4	27.4	27.4
		**RII**	32.6	26.5	26.4	28.4	26.9	24.5	28.1	19.2	27.7	27.4
		**RIII**	32.7	26.6	26.1	28.2	26.1	24.9	28.0	19.2	27.3	27.5
	**36'**	**RI**	30.0	26.2	25.2	28.3	25.8	22.9	26.9	18.7	26.1	26.9
		**RII**	29.1	26.1	25.2	28.1	25.7	22.9	26.5	18.3	26.5	26.7
		**RIII**	29.8	26.3	25.1	28.4	25.5	22	26.2	18.3	25.8	27.0

	**48'**	**RI**	31.0	26.0	25.8	28.7	26.1	24.1	27.2	18.7	26.2	27.4
		**RII**	31.1	26.2	25.3	28.5	26.1	24.8	27.1	19.2	26.1	27.2
		**RIII**	31.4	26.1	25.9	28.5	26.6	24.3	27	18.3	26.3	27.1
**Cold**	**0'**	**RI**	31.5	24.8	24.4	24.4	23.9	23.2	23.1	20.0	27.6	26.6
		**RII**	31.6	24.3	24.3	24.6	24.0	23.0	23.0	20.0	27.8	26.7
		**RIII**	31.5	24.3	24.6	24.2	23.8	22.8	23.1	20.1	27.0	26.4
	**12'**	**RI**	30.3	27.1	26.2	28.0	27.0	23.0	28.1	19.8	28.4	27.5
		**RII**	29.8	26.6	26.4	28.6	27.0	23.6	28.2	19.3	28.4	27.2
		**RIII**	30.5	27.9	26.0	28.1	27.0	23.0	28.2	19.7	28.6	27.7
	**24'**	**RI**	30.5	27.8	27.8	28.1	25.9	23.8	27.0	20.7	30.0	29.0
		**RII**	30.7	27.7	26.1	28.2	25.7	23.2	26.5	20.8	31.0	29.0
		**RIII**	30.3	28.0	27.0	28.0	25.1	24.0	27.4	20.4	30.4	30.0
	**36'**	**RI**	28.8	27.2	24.2	27.5	25.0	23.1	28.8	19.8	29.0	27.2
		**RII**	28.9	26.9	24.7	27.6	25.3	23.2	28.6	19.6	29.2	27.1
		**RIII**	28.8	27.3	23.9	27.4	25.0	23.2	28.8	19.9	29.2	27.4
	**48'**	**RI**	30.1	27.4	25.4	27.8	25.8	23.5	27.5	19.9	29.4	27.5
		**RII**	29.8	28.1	24.6	27.5	24.9	23.10	27.40	19.7	29.0	27.1
		**RIII**	29.7	27.0	25.0	27.6	25.0	24.00	26.80	20.0	29.0	27.3
**Hot**	**0'**	**RI**	31.5	24.2	24.4	24.4	23.8	22.9	23.1	20.0	26.6	26.6
		**RII**	31.4	24.3	24.4	24.6	23.8	23.2	23.0	20.0	26.8	27.1
		**RIII**	31.6	24.2	24.3	24.2	24.0	24.0	23.0	20.4	26.4	26.0
	**12'**	**RI**	31.5	22.9	26.1	29.5	25.4	24.0	27.1	18.9	27.3	27.2
		**RII**	31.7	22.8	26.2	29.2	25.4	24.1	27.0	18.6	27.6	27.8
		**RIII**	31.4	23.1	26.2	28.6	25.5	24.0	27.1	19.0	27.1	28.0
	**24'**	**RI**	33.3	24.2	28.2	29.1	25.8	25.5	27.0	20.0	27.6	27.2
		**RII**	33.2	24.1	27.8	29.1	25.4	25.1	26.4	19.8	27.2	27.4
		**RIII**	32.8	24.1	28.6	28.5	25.7	25.8	26.1	20.2	27.6	26.7
	**36'**	**RI**	31.7	23.3	24.9	26.1	23.6	23.9	24.0	19.3	25.4	25.3
		**RII**	31.7	23.4	25.1	26.1	23.9	24.0	24.0	19.2	25.6	26.4
		**RIII**	30.6	23.1	25.0	26.1	23.4	23.8	24.1	19.3	26.5	26.7

	**48'**	**RI**	31.9	23.7	25.2	27.1	25.4	24.2	25.1	20.0	26.1	27.1
		**RII**	30.7	23.1	24.8	26.3	25.3	24.8	24.8	20.2	26.6	26.7
		**RIII**	30.6	23.4	25.0	26.6	24.8	24.0	24.1	20.0	25.3	27.0
